# Exploring the selectivity of guanine scaffold in anticancer drug development by computational repurposing approach

**DOI:** 10.1038/s41598-021-95507-4

**Published:** 2021-08-10

**Authors:** D. R. Sherin, T. K. Manojkumar

**Affiliations:** School of Digital Sciences, Kerala University of Digital Sciences, Innovation and Technology, Trivandrum, Kerala India

**Keywords:** Computational chemistry, Target identification

## Abstract

Drug repurposing is one of the modern techniques used in the drug discovery to find out the new targets for existing drugs. Insilico methods have a major role in this approach. We used 60 FDA approved antiviral drugs reported in the last 50 years to screen against different cancer cell receptors. The thirteen compounds selected after virtual screening are analyzed for their druggability based on ADMET parameters and found the selectivity of guanine derivatives—didanosine, entecavir, acyclovir, valganciclovir, penciclovir, ganciclovir and valacyclovir as suitable candidates. The pharmacophore model, AARR, suggested based on the common feature alignment, shows that the two fused rings as in guanine and two acceptors-one from keto-oxygen (A5) and other from the substituent attached to nitrogen of imidazole ring (A4) give the druggability to the guanine derivatives. The NBO analysis on N9 is indicative of charge distribution from the ring to substituents, which results in delocalization of negative character in most of the ligands. The molecular dynamics simulations also pointed out the importance of guanine scaffold, which stabilizes the ligands inside the binding pocket of the receptor. All these results are indicative of the selectivity of guanine scaffold in anticancer drug development, especially as PARP1 inhibitors in breast, ovarian and prostate cancer. As these seven molecules are already approved by FDA, we can safely go for further preclinical trials.

## Introduction

Cancer, one of the foremost causes of mortality and morbidity worldwide, is a collective form of diseases with loss of control on growth and division of cells^1,2^. The existing treatments for this condition involves surgical removal of the growth with radiation and chemotherapies^3^. The key challenges of chemotherapy are the recurrence of the disease and severe side effects, which spoil the quality of life of the patient. In spite of its demerits, chemotherapy is still one of the broadly used method in treating all classes and stages of cancer progression^1^. Even though there are many heterocyclic compounds commercially available as anticancer agents, it still faces many challenges such as lack of specific targeting^4^. From drug discovery through FDA approval, developing a new medicine takes at least 10 years on average and costs an average of $2.6 billion^5^. Repurposing existing drugs that may have unanticipated effects as potential candidates is one way to resolve this barrier^6^. Revaluating the prevailing drugs through drug repurposing holds the potential to counterpart traditional drug discovery by modifying the high economic and time related costs and risks as many compounds have demonstrated safety in humans, it often negates the need for phase I clinical trials^7^. Also it has other advantages, such as implicit knowledge of its toxicity profile, drug metabolism, pharmacokinetics, and drug interactions^8^.

Repurposing approaches can be basically of two types- experimental screening approaches and in silico approaches. In silico methods apply sophisticated systematic approaches to existing data to identify new potential relations between drug and infection^9^. There are few recent reports on successful drug repurposing. Recently, Lorenzo et al. reported the repurposing effect of salicylanilide, anthelmintic drugs, in adenovirus infections^10^. Recognition of prostaglandin E2 as an activator of blood stem cell production and shows long-term safety in preclinical non-human primate transplant models^11^. White et al. reports the melanoma inhibiting capacity of leflunomide, an oral anti-lymphocyte agent that has been approved by the Food and Drug Administration (FDA) since 1998 for treatment of rheumatoid arthritis^12^. One of the initial reports toward the computer-aided drug repositioning for DNMT1 inhibitors is the work of Méndez-Lucio et al. that identified olsalazine as a novel hypomethylating agent^13^. The identification of the antiviral drug ribavirin as inhibitor of histone methyltransferase zeste homolog 2 (EZH2) is another example of computer aided drug repurposing^14^. Shaimerdenova et al. reported the effect of antiviral treatment in breast cancer cell lines. They reveal that acyclovir inhibits colony formation ability, diminishes the proliferation rate of cells and cell invasion capacity of the cancer cells^15^.

Molecular docking, dynamics and other computational tools are used to verify the drug and target interactions to envisage the potentiality of a drug or a ligand using mathematical calculations^16^. We applied inverse docking approach to identify the anticancer leads from antiviral drug database. As part of this, we selected 60 FDA approved small molecules over the past 50 years and screened them against 12 selected anticancer targets-EGFR, ABL, HSP90, CDK4/6, BRAF-wild and mutant, MEK1, BCL2, PARP, CMET and VEGF^17–34^. The best 7 compounds were screened based on druggability profile predicted by QikProp. The general features of the compounds were analyzed by pharmacophore modeling and electrostatic potential analysis. The results pointed out the selectivity of guanine moiety in anticancer drug development.

## Methodology

The protein preparation, receptor grid generation, ligand conformation generation, ADMET screening, pharmacophore modeling, molecular docking and dynamics were done by Schrodinger suite (2018-2)^35^. For this protein preparation wizard, LigPrep, QikProp, Phase-Pharm, Glide XP docking and Desmond tools were used in Maestro 11.2 interface in OPLS-2005 force field^36^. All the guanine derivatives were optimized and their HOMO–LUMO calculations were done by Density Functional Theory (DFT) using Gaussian 09 software packages and Chemcraft software was used for visualization purpose^37,38^. Poisson-Boltzmann electrostatic potential (ESP)was generated by Schrodinger^39^.

### Receptor grid generation

The crystal structures of all the selected receptors- epidermal growth factor receptor, EGFR (1M17); human proto-oncogene tyrosine-protein kinase ABL1, ABL kinase (3CS9); heat shock protein HSP 90-alpha, HSP90(5LNZ); cyclin dependent kinase 4, CDK4(3G33); cyclin dependent kinase 6 CDK6(3NUP); serine/threonine-protein kinase B-raf, BRAF wild(5VAM); mitogen-activated protein kinase kinase 1, MEK1(4U80); apoptosis regulator, BCL2(4AQ3); poly [ADP-ribose] polymerase 1, PARP(3L3M); B-Raf Kinase V600E oncogenic mutant, BRAF(3OG7); hepatocyte growth factor receptor, CMET(4MXC) and vascular endothelial growth factor, VEGF(1FLT) were retrieved from RCSB Protein Data Bank (PDB)^40^. The PDBs were processed, modified and refined before grid generation using protein preparation wizard of Schrodinger 2018-2. The grids were generated around the workspace ligand except in 3G33 and 1FLT, in which the sitemap analysis helped to identify the binding pocket. The three-dimensional grid space around the best binding pocket were used for further docking purpose.

### Ligand preparation

The three-dimensional structures of 60 FDA approved antiviral drugs reported in the last 50 years were collected from PubChem^41^. The structures were imported to maestro workspace to generate the optimized geometry and conformers using Ligprep tool. The best conformers were selected for docking and further analysis.

### QikProp analysis and ADMET prediction

The output of the Ligprep was used to analyze ADME/T(Absorption, Distribution, Metabolism, Excretion and Toxicity) properties using QikProp tool. The druggability of the compounds were predicted based on the parameters already defined by the software. The important parameters we selected are: #stars (few stars-more drug-like): 0 to 5; M.W. (Molecular Weight):130.0 to 725.0; HBA(Hydrogen bond acceptor): 2.0 to 20.0; HBD(hydrogen bond donor): 0.0 to 6.0; CNS (Central Nervous System activity): − 2 to + 2; FISA(hydrophilic component of the SASA on N, O, and H on heteroatoms) 7.0 to 330.0; QPlogS (Aqueous solubility): − 6.5 to 0.5; QPlogPo/w(octanol/water partition coefficient): − 2.0 to 6.5; QPlogKhsa (binding to human serum albumin): − 1.5 to 1.5; HOA (human oral absorption): 1 low, 2 medium, 3 hig ; Ro5 (Number of violations of Lipinski's rule of five): maximum is 4.

### Molecular docking

The optimized conformers of the 60 ligands were docked against the grid generated using extra precision mode (XP). The flexible docking of the ligands with the active binding sites were generated and the best poses corresponding to the interaction were defined based on Glide and Dock score. These scores were used to arrange the ligands on the basis of interactions. Here we compared the D-scores and − 6 kcal/mol was used as the standard maximum for comparison of inhibiting power.

### MM-GBSA for binding free energy prediction

The relative binding free energy (kcal/mol) of the selected ligands were calculated by MM-GBSA method by using Small-Molecule Drug Discovery Suite 2018-2. The more negative value indicates stronger binding as the MM-GBSA are approximate binding energies. It also generates a lot of energy properties like energies of the ligand, receptor, and the complexes as well as the energy differences relating to strain and binding,

Prime MMGBSA ΔG(bind), the binding free energy, is calculated with the equation:

$$\Delta {\text{G}}_{{{\text{bind}}}} = {\text{ E}}_{{\text{complex(minimized)}}} {-} \, \left\{ {{\text{E}}_{{\text{ligand(minimized)}}} + {\text{ E}}_{{\text{receptor(minimized}}} )} \right\}$$

### Molecular dynamics

The best scored receptor-ligand complexes were selected for molecular dynamics (MD) simulations, which predict the suitability and stability of the binding mode by integrating Newton’s equation of motion. The cubic box was defined around the selected complexes, solvate them with TIP4P water molecules and counter ions were added to neutralize the system. The energy minimized and equilibrated systems were further used to perform MD simulations for a period of 100 ns by using the Desmond module of the Schrödinger with an OPLS-2005 force field by setting NPT ensemble at 300 K and 1 atm pressure. Root mean square deviation (RMSD) plots for the backbone atoms for both proteins and the ligands were analyzed to predict the stability of binding. The protein–ligand (P–L) interaction diagram and histogram help us to define the interacting residues and their selectivity.

### Pharmacophore modeling

A pharmacophore model of guanine derivatives based on atleast 50% match seven-point hypothesis was generated to define the pharmacophore features of the ligands in common. The seven compounds were aligned based on common features-acceptor (A), donor (D), hydrophobic (H), negative ionic (N), positive ionic (P) and aromatic ring (R) and a model was generated based on Phase-Hypo score.

### Optimization and electrostatic potential

All the guanine derivatives were optimized using density functional theory (DFT) by applying Becke, 3-parameter, Lee–Yang–Parr (B3LYP) functional 6-311++G** basis set. The electrostatic potential (ESP) is generated by solving the Poisson–Boltzmann equations, with the partial charges of all the atoms in the structure in the workspace.

## Results and discussion

### Molecular docking and binding pose analysis

**Figure 1:**
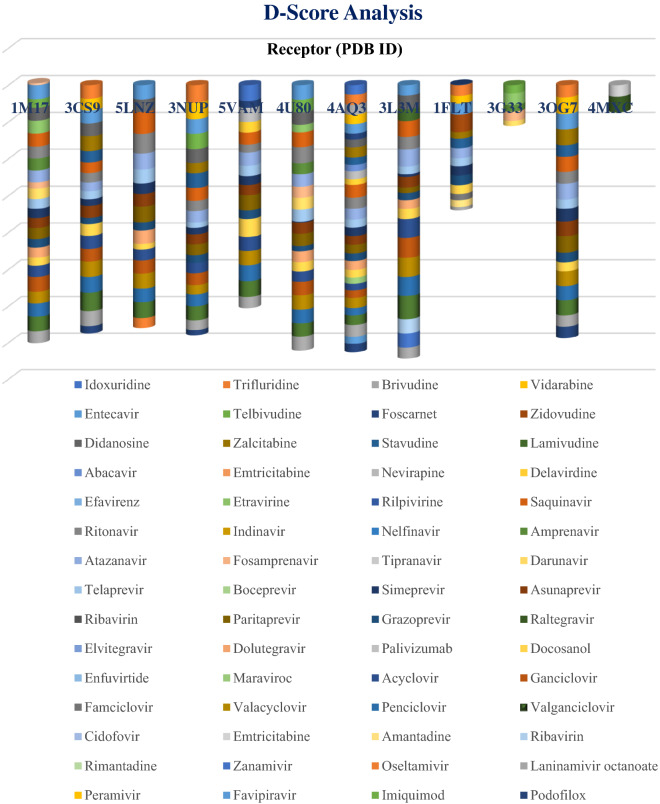
D-score analysis of sixty FDA approved drugs against selected twelve anticancer targets. (Each cylinder corresponds to a particular protein, represented by its PDB ID and the different ligands are represented by color codes as given under the plot. The D-score value of the ligands in a receptor are comparable to the width of the color band).

**Figure 2:**
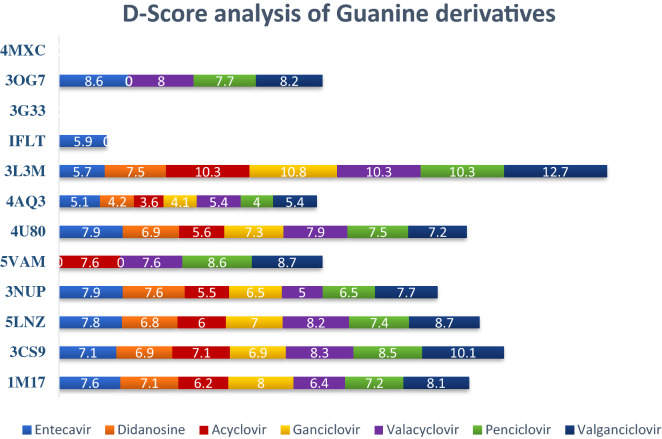
D-score analysis of seven guanine derivatives with twelve receptors (The color codes for the seven ligands are given under the figure).

The 60 selected FDA approved antiviral drugs reported during the last 50 years were docked against the 12 receptors existing in different cancer cell proliferations. The result analyzed on the basis of D-score (Figs. 1 and 2) shows that 13 compounds—entecavir, didanosine, saquinavir, ritonavir, atazanavir, asunaprevir, paritaprevir, acyclovir, ganciclovir, valacyclovir, penciclovir, valganciclovir and laninamivir octanoate have better binding affinity with more than 5 receptors. Among them the 7 guanine derivatives—entecavir, didanosine, acyclovir, ganciclovir, valacyclovir, penciclovir and valganciclovir are on the top of the profile. Entecavir shows better binding affinity with 1M17 (− 7.6 kcal/mol), 3CS9 (− 7.1 kcal/mol), 5LNZ (− 7.8 kcal/mol), 3NUP (− 7.9 kcal/mol), 4U80 (− 7.9 kcal/mol) and 3OG7 (− 8.6 kcal/mol). Didanosine and ganciclovir shows better binding with 1M17 (− 7.1 & − 8.0 kcal/mol), 3CS9 (− 6.9 & − 6.9 kcal/mol), 5LNZ (− 6.8 & − 7.0 kcal/mol), 3NUP (− 7.6 & − 6.5 kcal/mol), 4U80 (− 6.9 & − 7.3 kcal/mol) and 3L3M (− 7.5 & − 108 kcal mol) respectively while acyclovir shows maximum affinity with 1M17 (− 6.2 kcal/mol), 3CS9 (− 7.1 kcal/mol), 5LNZ (− 6.0 kcal/mol), 5VAM (− 7.7 kcal/mol) and 3L3M (− 10.3 kcal/mol). Penciclovir and valganciclovir shows healthier affinity with 1M17 (− 7.2 & − 8.1 kcal/mol), 3CS9 (− 8.5 & − 10.1 kcal/mol), 5LNZ (− 7.4 & − 8.7 kcal/mol), 3NUP (− 6.5 & − 7.7 kcal/mol), 5VAM (− 8.6 & − 8.7 kcal/mol), 4U80 (− 7.5 & − 7.2 kcal/mol), 3L3M (− 10.3 & − 12.7 kcal/mol) and 3OG7 (− 7.7 & − 8.2 kcal/mol) respectively whereas valacyclovir is better fitted in the binding pockets of 1M17 (− 6.4 kcal/mol), 3CS9 (− 8.3 kcal/mol), 5LNZ (− 8.2 kcal/mol), 5VAM (− 7.6 kcal/mol), 4U80 (− 7.9 kcal/mol), 3L3M (− 10.3 kcal/mol) and 3OG7 (− 8.0 kcal/mol). Valganciclovir shows better affinity with BRAF wild and mutant.

Penciclovir and valganciclovir shows better binding with 8 receptors and the second one is the best among the seven based on D-score. Thus, we can propose all the seven guanine derivatives for further inhibiting studies. All the seven guanine derivatives were suitably docked inside the binding pockets of 1M17, 3CS9 and 5LNZ. Even though 3L3M-entecavir affinity is little bit less, all the others show maximum affinity with 3L3M. We cannot define any leads against 4MXC and 3G33 based on D-score while entecavir only shows affinity with 1FLT. Among the 12 receptors selected for this study, we are able to propose guanine-based inhibitors for 9 of them.

In epidermal growth factor receptor (1M17), imidazolyl nitrogen of valganciclovir act as hydrogen bond acceptor from Met769, hydroxy hydrogen bonded to polar Thr766 and ammonium ion linked to negatively charged Asp831. Ganciclovir, penciclovir, didanosine, valacyclovir and acyclovir form H-bond with Met769 while hydroxyl hydrogen acts as donor to Thr766 in ganciclovir, penciclovir and acyclovir. In the case of entecavir, didanosine and valacyclovir forms bond with Asp831 whereas ganciclovir form hydrogen bond with Glu738 and Gln767. In entecavir also H-bond with Glu738 occurs and penciclovir form H-bond with Thr830. All these H-bonds give stability to all of the guanine derivative-1M17 complexes. Valganciclovir, penciclovir, valacyclovir and acyclovir are better inhibitors of 3CS9 than in 1M17 and valganciclovir being the best one here also. All the seven derivatives are strongly bound to the binding pocket of human ABL kinase, 3CS9. The hydrophobic interaction of guanine moiety with hydrophobic amino acid residues like Tyr253 and Phe382, and hydrogen bond interaction of hydroxyl groups are the major factors in stabilization of the complexes. In the case of valganciclovir, valacyclovir and didanosine, the 6-membered hydrophobic ring makes bond with Tyr253, while in penciclovir, acyclovir and entecavir, the hydrophobicity of 5-membered ring is utilized. Valacyclovir form hydrophobic interaction with Tyr253 by both the rings. Penciclovir, acyclovir and entecavir have hydrophobic interaction with both Tyr253 and Phe382 simultaneously. Even though, there are no such hydrophobic interactions in ganciclovir, the complex is stabilized by H-bond interaction of amino group from guanine with negatively charged Glu316.

**Figure 3:**
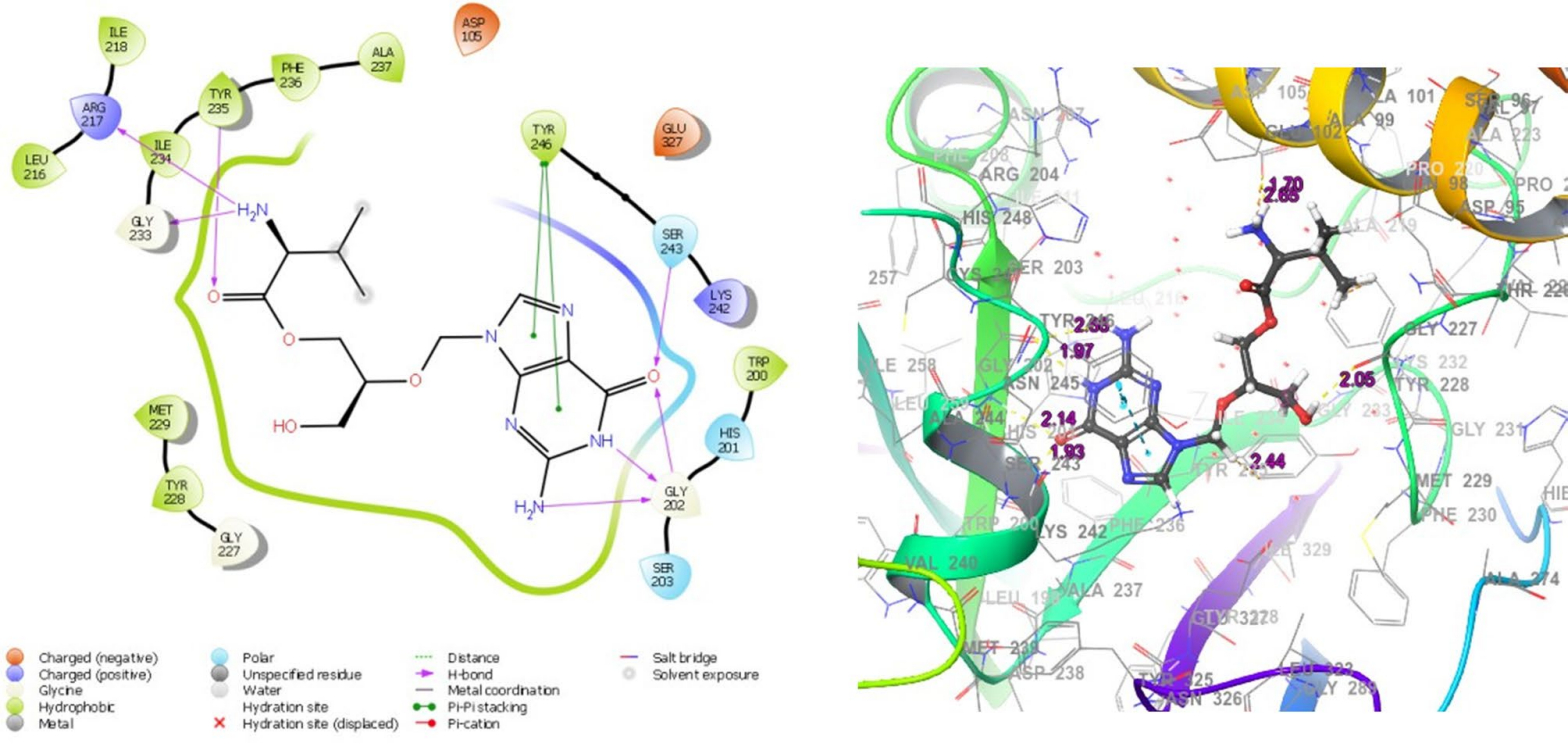
Binding poses of valganciclovir in poly [ADP-ribose] polymerase 1, PARP1(3L3M) (The 2D and 3D interaction diagrams from Schrodinger Maestro 11.2 GUI^36^, it gives the possible binding modes and the corresponding bond lengths).

The heat shock protein HSP 90-alpha (5LNZ) forms mainly H-bond interaction with hydrophobic and negatively charged residues. In valganciclovir, valacyclovir, ganciclovir didanosine and acyclovir, the -NH group from the 6-membered ring act as H bond donor for Asp93, Leu103 and Gly135. In other ligands, entecavir, penciclovir and acyclovir, the amino group attached to the cyclohexyl ring form H-bond with hydrophobic Tyr139, Leu107 and Leu103. The cyclin dependent kinase 6, CDK6 (3NUP) generally form H-bond with keto, amino and ring-NH group with negatively charged and hydrophobic residues. Val101 is one of the major residues that form H-bond. The mitogen-activated protein kinase kinase 1 (4U80) forms hydrogen bond with imino, amino and keto group of guanine scaffold, mainly using hydrophobic Met146, negatively charged Asp190 and Asp208 and polar Ser194. The other H-bonds are also due to Asp208 and Met146 in addition to Glu144, Ser194 and Lys97. Almost the seven ligands show best fitted in the binding pocket of poly [ADP-ribose] polymerase 1, PARP (3L3M), valganciclovir being the best with D-score and G-score of − 12.5 and − 12.7 kcal/mol respectively. On close observation of the binding pose (Fig. 3), it is found that the keto, imino and amino groups from the guanine scaffold strongly bonded to Gly202, with an extra bond between keto oxygen and polar Ser243. In addition, both the rings simultaneously form π-π stacking interaction with hydrophobic Tyr246. The hydroxyl and amino substituents on N9 also form H-bond with Tyr235, Arg217, Gly233, Gly227, Asp105, Tyr228, Tyr246, Met229, Lys242 and Glu327. In the case of entecavir, as the number of interactions is less and show lowest D-score and G-score. PARP is expressed mainly in breast, ovarian and prostate cancer, hence these drugs are being developed for chemotherapy in these types of malignancies.

Eventually, none of the guanine derivatives show better scores with 1FLT, 3G33 and 4MXC. Vascular epithelial growth factor receptor (1FLT) is a small receptor with a tiny binding pocket in which the guanine derivatives are not entrapped. The binding sites of cyclin dependent kinase 4 (3G33) and human Bcl-2 (4AQ3) are very small and none of the ligands penetrate into it. The binding pocket of hepatocyte growth factor receptor (4MXC) is almost tube like and it accommodates linear molecules very easily. So, the linear shaped arrangement of raltegravir is best fit inside the pocket of c-Met with D-score and G-score of − 8.8 and − 8.5 kcal/mol, which can be developed as a c-Met inhibitor. The serine/threonine-protein kinase B-raf (5VAM) and Braf fusion protein (3OG7) has a deep narrow binding pocket and the tube like ligand docosanol and paritaprevir penetrate deeply inside it with D-score and G-score of − 9.8 and − 9.0 kcal/mol respectively. The guanine derivatives valganciclovir, penciclovir, entecavir, acyclovir and valacyclovir also shows better binding while ganciclovir and didanosine did not give any strong binding poses which may be due to their comparatively small size.

### ADMET screening and binding free energy, ΔG_bind_

The QikProp analysis (Table 1) of 13 compounds shows that, only the 7 guanine derivatives-didanosine, entecavir, acyclovir, valganciclovir, penciclovir, ganciclovir and valacyclovir are best drugs based on zero violation from Lipinski’s rule of five and zero #stars. In all other cases druggability (#stars) is ≥ 4 with 2 or 3 violations from Ro5. The molecular weights of all compounds are within the range except for paritaprevir and asunaprevir. Hydrogen bond donors/acceptors, hydrophilic solvent accessible surface area from heteroatoms and central nervous system toxicity suggest the suitability of all thirteen drugs whereas oral absorption is best for didanosine and medium for other six guanine derivatives and saquinavir. The remaining five drugs-paritaprevir, asunaprevir, laninamivir, ritonavir and atazanavir shows minimum oral absorptive values. The octanol/water partition coefficient and human serum albumin binding are satisfied by all the thirteen ligands whereas the predicted aqueous solubility is good for all guanine derivatives and laninamivir and saquinavir. The binding free energies, ΔG_bind_, calculated by MM-GBSA analysis (Table 1) were less than − 42 kcal/mol in all the seven complexes, which indicates the strong binding of the selected ligands with the receptor PARP(3L3M).

**Table 1:** QikProp values of 13 ligands and their ΔG_bind_ (MM-GBSA) in PARP.

Compound	ΔGbind	#stars	M.W	HBA	HBD	CNS	FISA	QPlogS	QPlogPo/w	QPlogKhsa	HOA	Ro5
Didanosine	− 55.36	0	236.23	8.4	2.0	− 1	177.08	− 1.60	− 0.52	− 0.76	3	0
Entecavir	− 49.72	0	277.28	8.9	5.0	− 2	273.83	− 2.17	− 1.06	− 0.64	2	0
Acyclovir	− 48.98	0	225.21	8.9	4.0	− 2	244.44	− 2.31	− 1.55	− 0.95	2	0
Valganciclovir	− 52.53	0	354.36	11.9	6.0	− 2	239.62	− 0.05	− 1.42	− 0.81	2	0
Penciclovir	− 59.11	0	253.26	8.9	5.0	− 2	273.06	− 1.58	− 1.38	− 0.89	2	0
Valacyclovir	− 47.36	0	324.34	10.2	5.0	− 2	278.58	− 1.65	− 1.03	− 0.76	2	0
Ganciclovir	− 42.26	0	255.23	10.6	5.0	− 2	279.89	− 1.36	− 1.86	− 0.99	2	0
Paritaprevir	− 44.63	7	765.88	14.5	1.5	− 2	177.82	− 6.49	4.31	0.40	1	2
Asunaprevir	− 47.81	7	748.29	13.2	1.5	− 2	155.13	− 8.63	5.38	0.54	1	3
Laninamivir	− 52.17	4	472.54	12.6	7.0	− 2	306.13	− 1.55	0.13	− 1.08	1	2
Saquinavir	− 51.23	4	670.85	13.7	5.0	− 2	183.95	− 4.16	2.40	− 0.34	2	3
Ritonavir	− 44.91	6	720.94	10.9	3.2	− 2	128.85	− 6.72	5.91	0.57	1	3
Atazanavir	− 53.74	7	704.86	12.7	3.5	− 2	125.21	− 6.69	5.69	0.62	1	3

### Molecular dynamics

**Figure 4:**
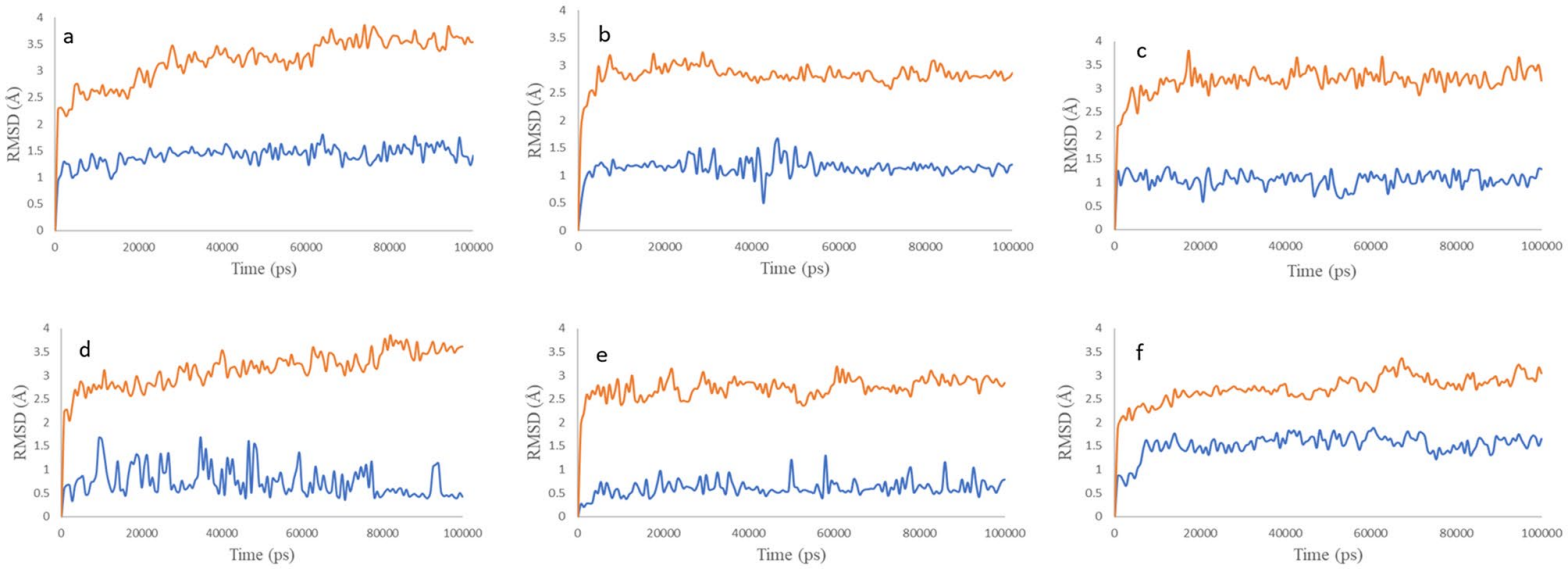
RMSD plots of the six top-scored complexes, (**a**–**f**) (3L3M-valganciclovir (**a**), 3L3M-ganciclovir (**b**), 3L3M-penciclovir (**c**), 4U80-entecavir (**d**), 3OG7-entecavir (**e**) and 5VAM-valganciclovir (**f**); the red colored graphs indicate the RMSD of proteins and the blue colored graphs indicate the RMSD of ligands inside the receptor).

**Figure 5:**
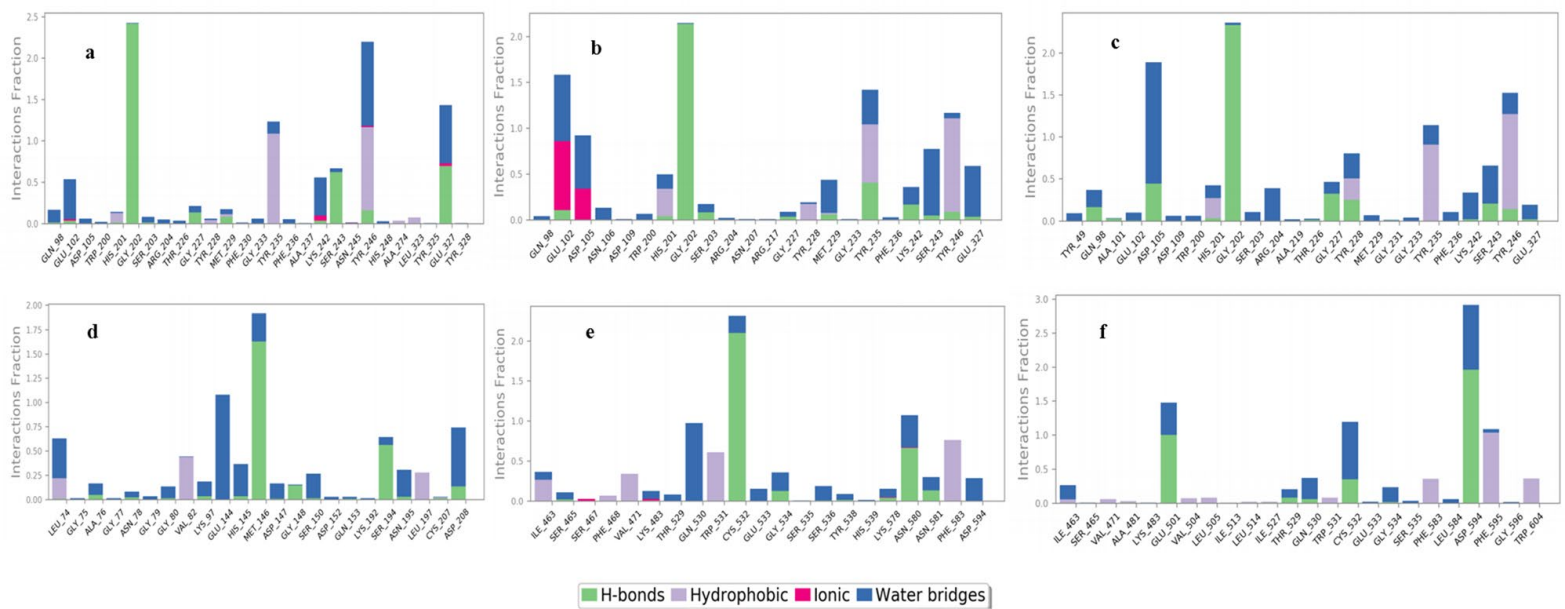
Protein–Ligand (P–L) interaction histograms of the six top-scored complexes, (**a**–**f**) [(3L3M-valganciclovir (**a**), 3L3M-ganciclovir (**b**), 3L3M-penciclovir (**c**), 4U80-entecavir (**d**), 3OG7-entecavir (**e**) and 5VAM-valganciclovir (**f**)].

To envisage the suitability and stability of docking poses, six top-scored complexes [3L3M-valganciclovir (**a**), 3L3M-ganciclovir (**﻿b**), 3L3M-penciclovir (**c**), 4U80-entecavir (), 3OG7-entecavir (**﻿e**) and 5VAM-valganciclovir (**﻿f**)] were selected and their MD simulations were carried out for 100 ns under OPLS-2005 forcefield. From the RMSD plots of the proteins and the ligands inside the binding pocket (Fig. 4), it is very clear that the proteins are stabilized under 3.5 Å and the ligands are stable under 1.5 Å in all the six cases without notable fluctuations. The protein–ligand (P–L) histogram (Fig. 5) and the interaction diagrams revealed that, in the case of **﻿a**, strong H-bonded interaction between –NH from pyrimidine and Gly202 lasts for 97% of the simulation time while the other two H-bonded interactions between -CO of guanine and Ser243 lasts for 62% and -CH-NH_2_ with Glu327 lasts for 69% of the simulation time. In addition, π–π stacking of imidazole and Tyr235 and Tyr246 lasts for 79%, also stabilizes the complex. When we analyze the data for the complex **﻿b**, it is very interesting that H-bonding of –NH from pyrimidine ring with Gly202 (98%), –OH with Tyr235(40%) and π–π stacking of imidazole with Tyr246 (88%) are the major stabilizing factors. The complex **﻿c** also forms H-bonded interaction of –NH from pyrimidine ring with Gly202 (100%) and π–π stacking interaction of imidazole with Tyr235 and Tyr246 (76%). In all these cases, Gly202, Tyr235 and Tyr246 are acting as the major points of interaction of ligands with 3L3M. The ligand entecavir inside 4U80 (**﻿d**) also shows strong H-bonded interaction with Met146 (100%) through the pyrimidine ring and Ser194 due to –OH (52%). In addition, hydrophobic interaction with Glu144 (86%) also stabilizes the complex. The H-bonded interactions of –NH from pyrimidine with Cys532 (95%) and –OH with Asn580 (37%) and π–π stacking interaction of the pyrimidine ring with Trp531 (42%) stabilizes the ligand entecavir inside the binding pocket of 3OG7 (**﻿e**). In the case of f also H-bonding formed between –CO and Asp594 (94%), –NH_2_ and Glu501 (100%), and π–π stacking of imidazole with Phe595 (57%) hold the ligand valganciclovir inside the binding site of 5VAM(**f**). From these analyses, we can conclude that the guanine moiety, specifically the -NH from pyrimidine and the –CO of guanine are act as the source for strong H-bonded interaction and the π–π stacking interaction.

### Pharmacophore modeling

**Figure 6:**
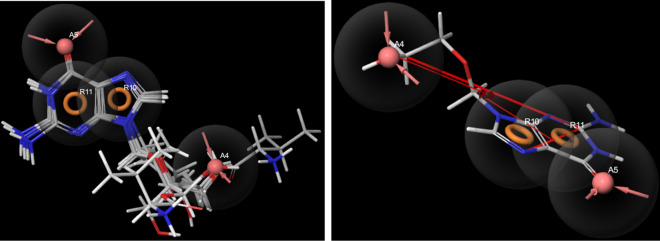
(**a**) The pharmacophore model (AARR) for guanine derivatives, (**b**) the dihedral angle between the guanine ring plane and the plane containing acceptor oxygen(A4) (generated by Maestro 11.2 GUI)^36^.

The pharmacophore model (Fig. 6) generated based on the common feature alignment, AARR shows that the two fused rings as in guanine and two acceptors-one from keto-oxygen (A5) and other from the substituent attached to nitrogen of imidazole ring (A4) give the druggability to the guanine derivatives. In valganciclovir and valacyclovir, A4 is from keto-oxygen while in others such as entecavir, didanosine, acyclovir, ganciclovir and penciclovir, the oxygen from –CH_2_–O–H act as the acceptor. The distance between imidazolyl-N and A4 (oxygen) varies from 4.42 to 5.02 Å. The dihedral angle between the ring plane and the plane containing oxygen (A4) is 15.0^o^.

### Electronic structure analysis (optimization/MESP/HOMO–LUMO)

**Figure 7:**
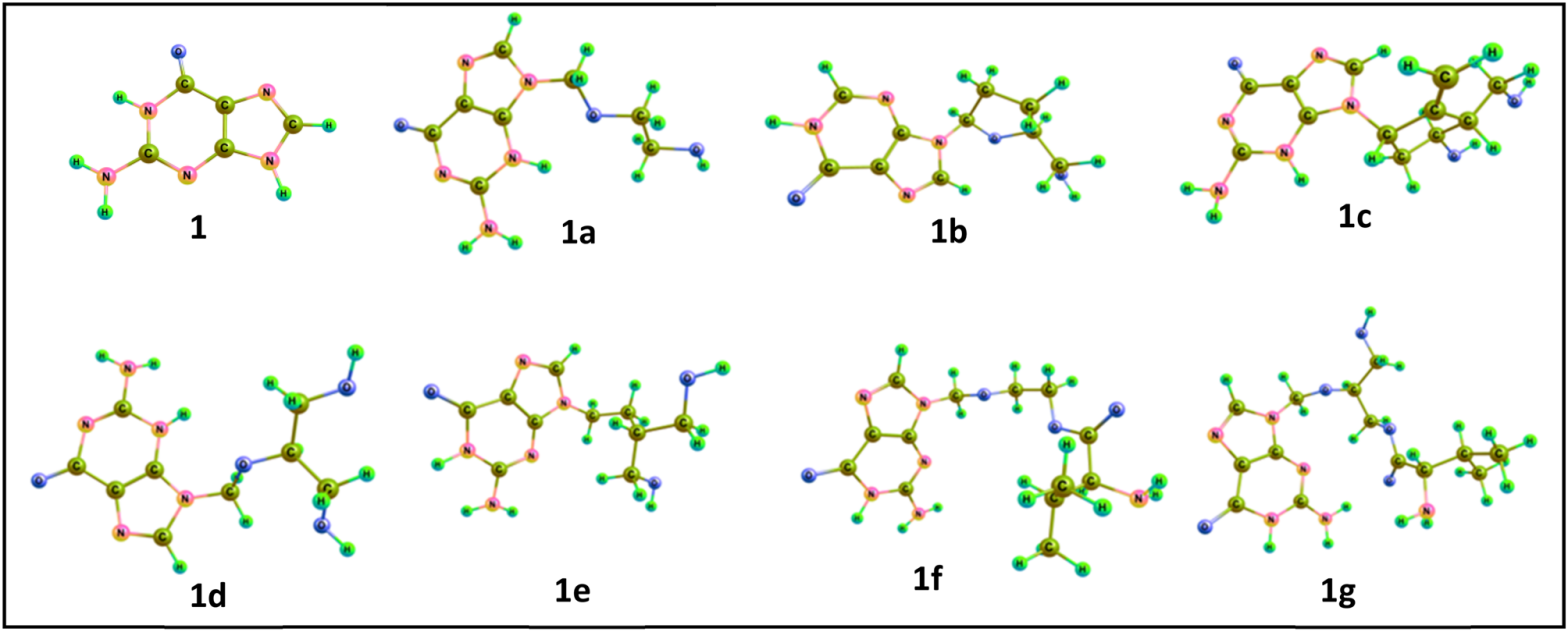
Optimized geometries (B3LYP/6-311++G**) of the guanine (**1**) and its seven derivatives—acyclovir (**1a**), didanosine (**1b**), entecavir (**1c**), ganciclovir (**1d**), penciclovir (**1e**), valacyclovir (**1f**) and valganciclovir (**1g**) (generated by Chemcraft 1.8 GUI)^37^.

**Figure 8:**
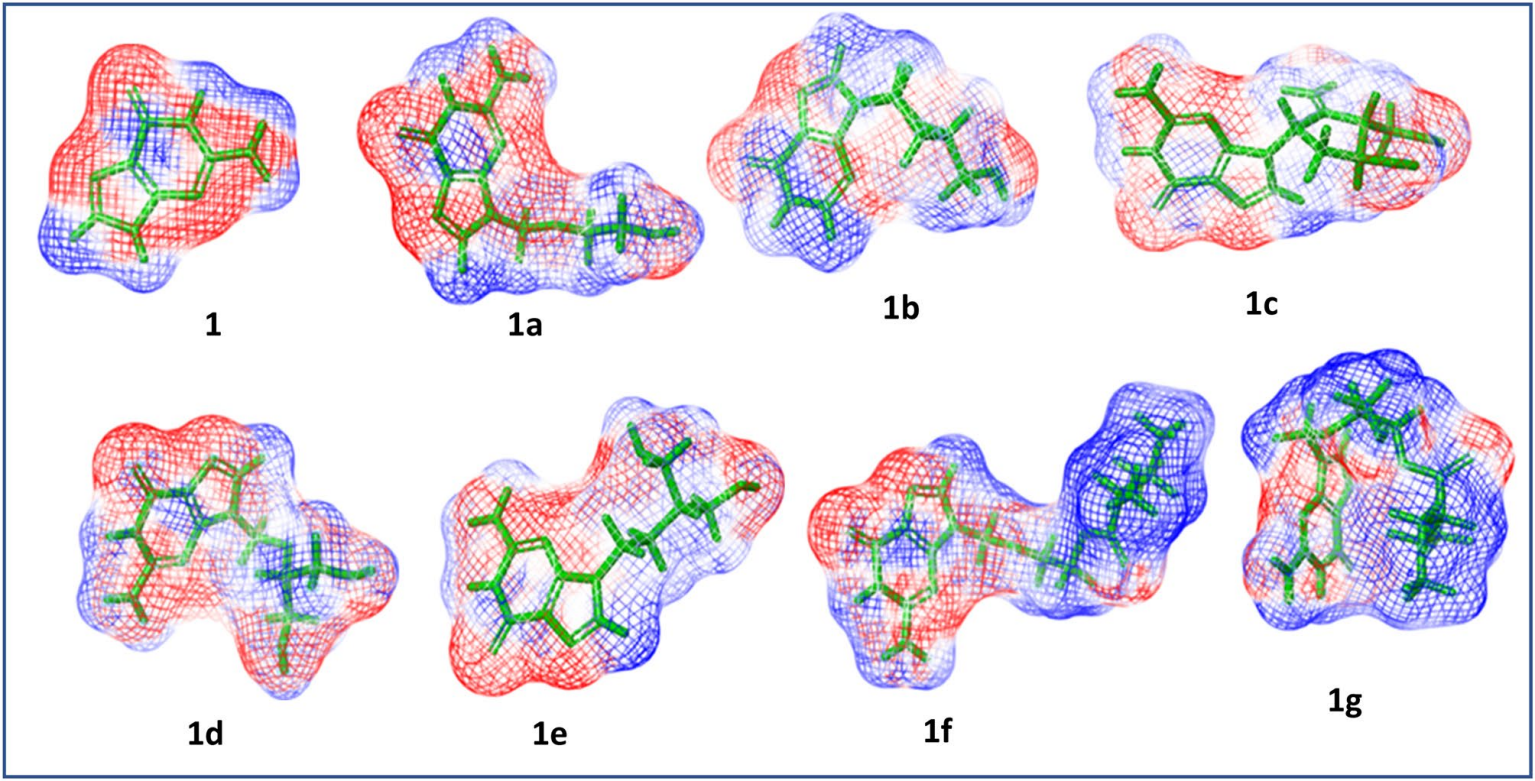
The Poisson-Boltzmann electrostatic potential maps (generated by Jaguar, Maestro 11.2 GUI)^36,39^ of guanine (**1**) and its seven derivatives—acyclovir (**1a**), didanosine (**1b**), entecavir (**1c**), ganciclovir (**1d**), penciclovir (**1e**), valacyclovir (**1f**) and valganciclovir (**1g**) (the electropositive and electronegative potential regions are indicated in blue and red color respectively).

Guanine and its seven derivatives-acyclovir, didanosine, entecavir, ganciclovir, penciclovir, valacyclovir and valganciclovir were optimized using density functional theory (Fig. 7; Supplementary Information, S1–S17) and their energies are tabulated below (Table 2). Even though guanine itself is planar and aromatic, the substitution changes its planarity and this bent structure help these molecules to entrap into the binding pocket of the receptors. The flexibility of valganciclovir by its linear and bent conformers makes it the most suitable inhibitor in almost all cases. The ESP analysis shows that all these compounds with common guanine scaffold share precise electronic properties (Fig. 8). Poisson–Boltzmann tool help to generate isosurfaces and a mapping to the molecular surface by using partial charges of all the atoms in the input structure. The ESP profiles shows that guanine scaffold is highly negatively charged (red colour), while the substituent tail is somewhat positive (blue colour) in valacyclovir and valganciclovir. In total the negative charge is delocalized within these molecules which helps in binding. The HOMO–LUMO energy gap (E_LUMO_–E_HOMO_) varies from 4.81 to 5.58 eV, which displays the relative activity of these compounds. The natural charge on N9 calculated by natural bond orbital (NBO) analysis indicate that the charge from the ring is distributed to substituents, which results in delocalization of negative character in most of the ligands. The natural charge on N9 in guanine is − 0.581 while on the derivatives, it ranges from − 0.432 to − 0.452.

**Table 2:** The optimized energy (B3LYP/6–311++G**, gas phase), natural charge on N_9_, E_LUMO_–E_HOMO_ (eV) and PARP interacting residues of guanine and its derivatives.

	Optimized energy B3LYP/6-311++G** (gas phase)	Natural charge on N_9_	E_LUMO_–E_HOMO_ (eV)	PARP1interacting residues
Guanine	− 542.71	− 0.581	5.15	–
Acyclovir	− 810.75	− 0.452	5.58	Ser243, Gly202, Tyr246, Tyr235, Arg217, Gly233
Didanosine	− 833.21	− 0.451	5.16	Ser243, Gly202, Tyr246, Asp105, tyr228
Entecavir	− 965.99	− 0.438	4.81	Ser243, Gly202, Tyr246, Met229, Gly227
Ganciclovir	− 925.69	− 0.446	5.18	Ser243, Gly202, Tyr246, Lys242, Glu327, Gly227
Penciclovir	− 889.81	− 0.432	5.06	Ser243, Gly202, Tyr246, Glu327
Valacyclovir	− 1137.20	− 0.443	5.18	Ser243, Gly202, Tyr246, Glu327, Lys242
Valganciclovir	− 1251.76	− 0.443	5.16	Ser243, Gly202, Tyr246

## Conclusions

We effectively used virtual screening for predicting the activity of antiviral drugs as inhibitors of selected cancer targets. The sixty FDA approved drugs from the last 50 years were selected and screened against twelve receptors- epidermal growth factor receptor, EGFR (1M17); human proto-oncogene tyrosine-protein kinase ABL1, ABL kinase (3CS9); heat shock protein HSP 90-alpha, HSP90(5LNZ); cyclin dependent kinase 4, CDK4(3G33); cyclin dependent kinase 6 CDK6(3NUP); serine/threonine-protein kinase B-raf, BRAF wild(5VAM); mitogen-activated protein kinase kinase 1, MEK1(4U80); apoptosis regulator, BCL2(4AQ3); poly [ADP-ribose] polymerase 1, PARP(3L3M); B-Raf Kinase V600E oncogenic mutant, BRAF(3OG7); hepatocyte growth factor receptor, CMET(4MXC) and vascular endothelial growth factor, VEGF(1FLT) retrieved from RCSB Protein Data Bank (PDB). The D-score analysis shows that 13 compounds—entecavir, didanosine, saquinavir, ritonavir, atazanavir, asunaprevir, paritaprevir, acyclovir, ganciclovir, valacyclovir, penciclovir, valganciclovir and laninamivir octanoate have better binding affinity with more than 5 receptors. The stability of these complexes were confirmed by MD simulations, which focuses the importance of guanine moiety in all the screened cases. The QikProp analysis of these 13 compounds indicate that, only the 7 guanine derivatives—didanosine, entecavir, acyclovir, valganciclovir, penciclovir, ganciclovir and valacyclovir are best drugs based on zero violation from Ro5 and zero #stars. The results suggest that compounds can be developed as chemotherapeutic agents, specifically as PARP1 inhibitors in breast, ovarian and prostate cancer. The pharmacophore model suggested based on the common feature alignment, AARR shows that the two fused rings as in guanine and two acceptors-one from keto-oxygen (A5) and other from the substituent attached to nitrogen of imidazole ring (A4) give the druggability to the guanine derivatives. The NBO analysis on N9 is indicative of charge distribution from the ring to substituents, which results in delocalization of negative character in most of the ligands. As these molecules are already approved by FDA as antiviral drugs, we can directly go for preclinical and clinical studies of them against different cancer cell proliferations. As this is a pure computational repurposing approach, wet lab experiments are essential for the cross validation of the results. We can expand this type of drug repurposing protocol along with application of artificial intelligence and in vitro validation for finding out suitable inhibitors for other ailments in near future.

## Supplementary Information

Supplementary Information.
