# Supplementary material for: Exploring the selectivity of guanine scaffold in anticancer drug development by computational repurposing approach
Source: Sci Rep. 2021 Aug 10;11:16251. doi: 10.1038/s41598-021-95507-4 (PMC8355261; doi:10.1038/s41598-021-95507-4)
Supplement: Supplementary file 1 — Supplementary Information. [file 41598_2021_95507_MOESM1_ESM.docx]

**Supplementary Information**

**Exploring the Selectivity of Guanine Scaffold in Anticancer Drug Development by Computational Repurposing Approach**

D. R. Sherin* and T. K. Manojkumar*

*School of Digital Sciences*

*Kerala University of Digital Sciences, Innovation and Technology, Trivandrum, Kerala, India.*

*Email: [sherin.dr@iiitmk.ac.in](mailto:sherin.dr@iiitmk.ac.in), [manojtk@iiitmk.ac.in](mailto:manojtk@iiitmk.ac.in)

**Standard orientations of Guanine derivatives (B3LYP/6-311++G**)**

1. Acyclovir

---------------------------------------------------------------------

Center Atomic Atomic Coordinates (Angstroms)

Number Number Type X Y Z

---------------------------------------------------------------------

1 8 0 -2.028849 -0.429951 -0.010958

2 8 0 4.408371 -0.129730 -0.617490

3 8 0 -5.474167 0.739364 -0.334969

4 7 0 0.040213 -1.487460 0.278252

5 7 0 0.525465 0.952290 0.353175

6 7 0 2.124175 -2.225752 -0.249788

7 7 0 2.829033 1.505796 -0.070918

8 7 0 1.273664 3.189267 0.364827

9 6 0 0.860446 -0.378772 0.189089

10 6 0 -1.348515 -1.508800 0.690819

11 6 0 2.140563 -0.825057 -0.131569

12 6 0 0.874213 -2.594890 -0.010406

13 6 0 3.232650 0.150351 -0.306012

14 6 0 1.594719 1.855545 0.206850

15 6 0 -3.474255 -0.387418 0.243387

16 6 0 -4.053740 0.686896 -0.670931

17 1 0 -1.441752 -1.347630 1.777787

18 1 0 -1.777789 -2.486171 0.430020

19 1 0 0.496583 -3.606177 -0.020868

20 1 0 -0.450791 1.229038 0.322616

21 1 0 -3.673425 -0.138716 1.296155

22 1 0 -3.928017 -1.362093 0.014175

23 1 0 -3.561414 1.652076 -0.477157

24 1 0 -3.892004 0.410402 -1.722364

25 1 0 0.409997 3.498074 0.786075

26 1 0 2.031071 3.846196 0.240096

27 1 0 -5.959641 1.341425 -0.935394

---------------------------------------------------------------------

1. Didanosine

---------------------------------------------------------------------

Center Atomic Atomic Coordinates (Angstroms)

Number Number Type X Y Z

---------------------------------------------------------------------

1 8 0 2.248772 0.140403 -0.935868

2 8 0 4.669656 -1.376176 -0.303966

3 8 0 -4.238633 -1.730639 0.476612

4 7 0 -0.040244 -0.015417 -0.434117

5 7 0 -1.164304 -1.927676 -0.057900

6 7 0 -1.868624 1.593807 -0.297871

7 7 0 -3.913824 0.520966 0.168214

8 6 0 1.071611 0.897515 -0.804921

9 6 0 1.346025 1.953967 0.273252

10 6 0 2.385359 1.253902 1.155002

11 6 0 3.188334 0.447925 0.130120

12 6 0 3.804073 -0.822640 0.679964

13 6 0 -1.355900 0.332006 -0.245858

14 6 0 0.002330 -1.391619 -0.324170

15 6 0 -2.031172 -0.865092 -0.005101

16 6 0 -3.453385 -0.836194 0.240393

17 6 0 -3.150791 1.621664 -0.085837

18 1 0 0.814200 1.332856 -1.772619

19 1 0 0.441222 2.264231 0.791639

20 1 0 1.780458 2.833941 -0.208894

21 1 0 3.011435 1.951964 1.712256

22 1 0 1.898200 0.586277 1.872967

23 1 0 3.987341 1.054978 -0.310736

24 1 0 3.006508 -1.524507 0.954791

25 1 0 4.362660 -0.563329 1.591116

26 1 0 0.924316 -1.928929 -0.469684

27 1 0 4.989697 -2.229706 0.002464

28 1 0 -3.676139 2.570990 -0.105873

29 1 0 -4.908173 0.632177 0.322998

---------------------------------------------------------------------

1. Entecavir

---------------------------------------------------------------------

Center Atomic Atomic Coordinates (Angstroms)

Number Number Type X Y Z

---------------------------------------------------------------------

1 8 0 -3.437174 1.627557 -1.655105

2 8 0 -5.217792 -0.444497 -0.708995

3 8 0 4.318800 -2.030552 -0.978949

4 7 0 0.254465 -0.304004 0.309609

5 7 0 2.236066 1.175705 0.439429

6 7 0 1.295515 -2.148347 -0.452454

7 7 0 4.277957 0.131176 -0.212798

8 7 0 4.231632 2.376251 0.380758

9 6 0 -0.800650 0.641075 0.714203

10 6 0 -3.235286 0.251212 0.406622

11 6 0 -2.609180 0.752718 -0.915009

12 6 0 -1.345849 1.484660 -0.459834

13 6 0 -2.038495 -0.063796 1.287820

14 6 0 -4.216258 -0.897887 0.213902

15 6 0 1.579610 -0.002788 0.151388

16 6 0 -2.024466 -0.800485 2.394918

17 6 0 0.152162 -1.634918 -0.093831

18 6 0 2.218525 -1.132632 -0.302808

19 6 0 3.663256 -1.106665 -0.545912

20 6 0 3.609635 1.153603 0.191450

21 1 0 -0.352493 1.277179 1.482707

22 1 0 -3.785309 1.101670 0.832933

23 1 0 -2.332020 -0.116749 -1.531185

24 1 0 -0.619366 1.618775 -1.262808

25 1 0 -1.643146 2.474559 -0.099456

26 1 0 -3.692695 -1.773524 -0.190390

27 1 0 -4.679078 -1.181623 1.165853

28 1 0 -2.918361 -1.277153 2.780939

29 1 0 -1.110200 -0.962572 2.955816

30 1 0 -4.278584 1.171837 -1.791497

31 1 0 -0.793718 -2.150652 -0.075180

32 1 0 1.755183 2.063310 0.399405

33 1 0 -5.825650 -1.163978 -0.904271

34 1 0 3.941815 2.913239 1.186827

35 1 0 5.235484 2.305568 0.285437

---------------------------------------------------------------------

1. Ganciclovir

---------------------------------------------------------------------

Center Atomic Atomic Coordinates (Angstroms)

Number Number Type X Y Z

---------------------------------------------------------------------

1 8 0 1.623721 -0.099988 0.177135

2 8 0 3.878238 -1.934244 -0.376696

3 8 0 4.471804 2.105905 0.241578

4 8 0 -4.758931 -0.682711 0.684456

5 7 0 -0.314430 -1.253829 -0.334361

6 7 0 -1.233293 1.032746 -0.447231

7 7 0 -2.193260 -2.334212 0.259526

8 7 0 -3.556882 1.172531 0.070503

9 7 0 -2.318126 3.091957 -0.351300

10 6 0 2.980971 0.272527 -0.099413

11 6 0 1.050422 -1.033459 -0.733405

12 6 0 -1.310344 -0.323445 -0.228343

13 6 0 -2.460883 -0.982175 0.128253

14 6 0 -0.927108 -2.464808 -0.009067

15 6 0 3.979125 -0.751165 0.423181

16 6 0 3.168864 1.633606 0.569338

17 6 0 -3.695058 -0.218438 0.333085

18 6 0 -2.431088 1.716364 -0.236654

19 1 0 3.125376 0.384305 -1.182415

20 1 0 1.574672 -1.988922 -0.716167

21 1 0 1.087281 -0.618791 -1.750133

22 1 0 3.747776 -0.969535 1.473645

23 1 0 4.981630 -0.317334 0.358033

24 1 0 3.039474 1.528827 1.654060

25 1 0 2.393515 2.312888 0.195805

26 1 0 -0.360649 -3.384306 -0.001456

27 1 0 -0.355740 1.505923 -0.275438

28 1 0 4.565880 -2.552553 -0.112433

29 1 0 4.623868 2.946090 0.684007

30 1 0 -1.741139 3.434544 -1.107475

31 1 0 -3.218060 3.548789 -0.293620

---------------------------------------------------------------------

1. Penciclovir

---------------------------------------------------------------------

Center Atomic Atomic Coordinates (Angstroms)

Number Number Type X Y Z

---------------------------------------------------------------------

1 8 0 3.340323 2.267310 0.243009

2 8 0 3.777530 -1.730405 -1.237537

3 8 0 -4.088319 -1.439771 -1.108782

4 7 0 -0.055213 -0.826528 1.065226

5 7 0 -1.432234 -2.409911 0.250779

6 7 0 -1.321277 1.178279 0.536178

7 7 0 -3.335781 0.655157 -0.569933

8 7 0 -2.736892 2.881675 -0.164423

9 6 0 2.474239 0.124897 -0.449827

10 6 0 2.424533 -0.427415 0.985961

11 6 0 1.101248 -0.218788 1.730629

12 6 0 3.768163 -0.302036 -1.147679

13 6 0 2.299179 1.641811 -0.521315

14 6 0 -1.135560 -0.168937 0.536452

15 6 0 -1.976206 -1.162897 0.036715

16 6 0 -0.299931 -2.174358 0.859439

17 6 0 -3.209005 -0.779706 -0.594171

18 6 0 -2.439762 1.541169 -0.035296

19 1 0 1.648036 -0.312330 -1.024249

20 1 0 3.210210 0.048394 1.581182

21 1 0 2.657334 -1.494200 0.947148

22 1 0 0.866428 0.839683 1.842616

23 1 0 1.177516 -0.641491 2.736457

24 1 0 3.802792 0.142838 -2.151097

25 1 0 4.636316 0.058635 -0.584001

26 1 0 1.314000 1.925548 -0.135533

27 1 0 2.353008 1.962879 -1.570328

28 1 0 0.403356 -2.927320 1.182801

29 1 0 4.616527 -2.013677 -1.611827

30 1 0 3.225405 3.220883 0.202231

31 1 0 -4.157133 0.995931 -1.053106

32 1 0 -2.150605 3.478050 0.401061

33 1 0 -3.711061 3.145963 -0.167105

---------------------------------------------------------------------

1. Valacyclovir

---------------------------------------------------------------------

Center Atomic Atomic Coordinates (Angstroms)

Number Number Type X Y Z

---------------------------------------------------------------------

1 8 0 -2.728364 1.037199 0.617948

2 8 0 -0.130953 2.219729 0.170937

3 8 0 -4.627387 1.120504 -0.590887

4 8 0 4.889633 -1.605836 -1.651750

5 7 0 2.127287 1.749548 -0.087869

6 7 0 -5.235992 -1.566583 0.103916

7 7 0 3.443257 1.174837 -1.816602

8 7 0 2.395613 -0.191934 1.346153

9 7 0 3.826964 -1.790115 0.369520

10 7 0 2.803384 -2.200604 2.433109

11 6 0 -2.813113 -1.770191 -0.475713

12 6 0 -3.906825 -1.015520 0.338559

13 6 0 -3.077450 -1.724839 -1.987359

14 6 0 -2.682159 -3.214457 0.022138

15 6 0 -3.827796 0.488081 0.056872

16 6 0 1.161440 2.483151 0.681140

17 6 0 2.637965 0.506821 0.210742

18 6 0 -2.500286 2.436127 0.354683

19 6 0 -1.163092 2.814346 0.944141

20 6 0 3.453498 0.173308 -0.865494

21 6 0 2.648621 2.085226 -1.332039

22 6 0 4.143550 -1.088167 -0.847112

23 6 0 3.009372 -1.347618 1.374668

24 1 0 -1.869975 -1.250463 -0.277283

25 1 0 -3.662841 -1.150126 1.396808

26 1 0 -3.625139 -3.751462 -0.100763

27 1 0 -2.409598 -3.246040 1.081722

28 1 0 -3.992713 -2.265906 -2.244839

29 1 0 -1.905842 -3.743126 -0.538265

30 1 0 -2.253821 -2.198955 -2.527435

31 1 0 -3.170595 -0.701183 -2.361050

32 1 0 -5.612417 -1.212022 -0.770201

33 1 0 -5.878246 -1.269075 0.830362

34 1 0 1.243584 2.158139 1.723774

35 1 0 1.390079 3.554988 0.611784

36 1 0 -2.516520 2.612995 -0.722168

37 1 0 -3.298063 3.025406 0.814739

38 1 0 -1.102219 2.478826 1.988721

39 1 0 -1.072091 3.911190 0.929693

40 1 0 2.391384 3.019165 -1.809773

41 1 0 4.226593 -2.718934 0.414769

42 1 0 2.323112 -1.775819 3.212427

43 1 0 3.545962 -2.834008 2.688053

---------------------------------------------------------------------

1. Valganciclovir

---------------------------------------------------------------------

Center Atomic Atomic Coordinates (Angstroms)

Number Number Type X Y Z

---------------------------------------------------------------------

1 8 0 -0.475982 2.408944 0.156718

2 8 0 1.864624 0.642354 -0.263461

3 8 0 1.163953 4.803287 0.216765

4 8 0 1.700760 -1.272427 -1.444778

5 8 0 -4.631001 -2.594658 1.126790

6 7 0 -2.609080 1.435459 0.121088

7 7 0 2.670270 -2.754015 0.775237

8 7 0 -4.232873 0.516679 1.374958

9 7 0 -1.703081 -0.530425 -0.962903

10 7 0 -2.863481 -2.478988 -0.326287

11 7 0 -1.068044 -2.578623 -1.835619

12 6 0 4.294464 -0.857863 0.722192

13 6 0 2.800217 -1.303236 0.729237

14 6 0 0.633684 2.673806 -0.706896

15 6 0 1.187636 1.386103 -1.305879

16 6 0 4.960839 -1.219856 2.054720

17 6 0 5.070944 -1.443070 -0.465742

18 6 0 2.056718 -0.672091 -0.452659

19 6 0 -1.746438 2.428383 -0.467161

20 6 0 1.657452 3.471823 0.086003

21 6 0 -2.573542 0.084086 -0.128925

22 6 0 -3.587593 -0.465909 0.648169

23 6 0 -3.628860 1.623148 1.046864

24 6 0 -3.815524 -1.883491 0.576374

25 6 0 -1.877895 -1.829532 -1.024129

26 1 0 4.289339 0.233445 0.626075

27 1 0 2.349488 -0.890419 1.636165

28 1 0 0.312292 3.306927 -1.545191

29 1 0 1.911225 1.609525 -2.095720

30 1 0 0.383130 0.774078 -1.712251

31 1 0 5.132258 -2.532792 -0.395274

32 1 0 6.094141 -1.058204 -0.473795

33 1 0 4.928864 -2.298848 2.219716

34 1 0 4.457705 -0.733610 2.896049

35 1 0 6.006528 -0.899525 2.059688

36 1 0 4.613822 -1.189484 -1.426634

37 1 0 -2.219082 3.406864 -0.332911

38 1 0 -1.657498 2.212002 -1.535700

39 1 0 2.616008 3.467839 -0.449472

40 1 0 1.800114 2.996790 1.060582

41 1 0 2.767397 -3.148754 -0.154925

42 1 0 1.754157 -3.022566 1.116377

43 1 0 -3.863736 2.608047 1.423499

44 1 0 -2.984637 -3.478147 -0.433000

45 1 0 1.564775 5.215107 0.986829

46 1 0 -0.182043 -2.135093 -2.054830

47 1 0 -1.001660 -3.567601 -1.649883

---------------------------------------------------------------------
